# Congress of American Physicians and Surgeons

**Published:** 1888-10

**Authors:** 


### SOCIETY REPORTS.

### CONGRESS OF AMERICAN PHYSICIANS AND SURGEONS.

FIRST DAY—Morning Session.

The First Tri-ennial Meeting. Held in Washington, D. C., on September 18, 19, and 20.

The meeting for organization and to receive the report of the Executive Committee was held on September 18.

It was called to order by Professor WILLIAM PEPPER, of Philadelphia, as Chairman of the Executive Committee, who made a report of the work of that committee.

Dr. JOHN S. BILLINGS, of the United States Army, President of the Congress, then took the chair and responded in a brief address.

Dr. SAMUEL C. BUSEY, of Washington, Chairman of the Committee of Arrangements, welcomed the Congress, and dwelt upon the advantages which that city offers as a place of meeting for scientific societies.

The following are the by-laws adopted:

1. This organization shall be known as the Congress of American Physicians and Surgeons.

2. It shall be composed of national associations for the promotion of medical and allied sciences.

3. It shall hold its sessions tri-ennially in the city of Washington, D. C.

4. The officers of the Congress shall be a president, vice-president, a secretary, a treasurer, and an executive committee.

5. The president shall be elected by the executive committee, of which he shall ex officio be a member. He shall preside at the sessions of the Congress. He shall deliver an address.

6. The presidents of the participating societies shall be ex-officio the vice-presidents of the Congress.

7. The secretary and treasurer shall be elected by the executive committee. They shall be ex-officio members of the executive committee.

8. The executive committee shall be composed of one member from each participating society; and said member shall be elected by the various societies at the next annual meeting subsequent to the Congress. It shall be charged with all duties pertaining to the organization of and preparation for the ensuing Congress, including the election of all officers, and of a committee of arrangements. It shall superintend the publication of the transactions of the Congress.

9. The expenses of the Congress shall be divided between the participating societies in proportion to their membership.

10. The admission of new associations to participation in the Congress shall be by unanimous vote of the executive committee.

FIRST DAY—Evening Session.

Professor REGINALD H. FITZ, of Boston, read a paper on "The Treatment of Intestinal Obstruction," followed by one on the same subject by Professor N. SENN, of Milwaukee.

The subject of this paper was the diagnosis and medical treatment of the acute internal, mechanical varieties of intestinal obstruction. The only causes recognized were strangulation from adhesions, vitelline remains, peritoneal slits, pockets, and rings; intussusception, twists and knots, abnormal contents, strictures, and tumors. The evidence presented resulted from an analysis of 295 cases collected from medical literature since 1880.

Professor SENN first directed attention to irrigation of the stomach as practiced by Kussmaul. He said the mechanical effects of such a measure were limited to he segment of intestine below the ileo-cæcal valve. Experiment and clinical experience united in demonstrating the fact that the ileo-cæcal valve is impermeable to fluids. Why should we use fluid in attempting the mechanical correction of a mechanical cause below or above the ileo-cæcal valve? Again, all efforts at overcoming the ileo-cæcal valve by rectal injections result in serious injury to the distended colon. Why, said he, should we not for diagnostic, and more particularly for therapeutic, purposes, resort to the lightest, the most compressible, substance known—hydrogen gas, perfectly harmless, non-toxic, non-irritant? Experiment has shown by means of a manometer that a pressure of one-fourth of a pound to two pounds to the square inch is adequate to force hydrogen gas from anus to mouth; consequently the alimentary canal is permeable to hydrogen inflations. This means of investigation would show that it requires a pressure of from eight to twelve pounds to produce a palpable injury to any of the coats of the intestines; but when fluids are forced beyond the ileo-cæcal valve the surgeon invariably finds on post-mortem examination longitudinal rents in the serous coat. When the hydrogen gas is forced from anus to mouth no tangible injury has been produced anywhere.

Enterotomy, so frequently practiced in the past, he hoped had become obsolete. He meant Nelaton's operation. It is true, distinguished surgeons of to-day favor enterotomy in cases of acute intestinal obstruction in all instances where they are unable to ascertain the seat and character of the obstruction. They believe that, for the time being, an outlet for the accumulating intestinal contents places the intestinal canal in a favorable condition for subsequent radical measures.

Colotomy is indicated in all cases of intestinal obstruction below the ileo-cæcal region, where the obstruction has been caused by conditions beyond radical measures, or the general condition of the patient does not warrant the most serious operation of abdominal section.

Lumbar colotomy is an obsolete operation—in fact, scarcely any surgeon will attempt to make an extra peritoneal colotomy, in these days of safe abdominal operations. In cases, for instance, of cicatricial contraction, of harmless obstruction, unattended by indications of gangrene or perforation, the surgeon can safely make the obstruction harmless, and at the same time more efficient by resorting to enterostomy. Instead of spending an hour or an hour and a half in the process of suturing, as practiced in Europe, the surgeon can by a simple device unite the bowel above and below the seat of obstruction by approximation with perforated decalcified bone plates, more especially in cases of inoperable obstruction, where carcinoma and sarcoma have gone beyond their legitimate field and beyond the surgeon's reach.

DISCUSSION.

The PRESIDENT called upon Professor William Pepper, of Philadelphia, to open the discussion, but he declined in favor of Mr. Arthur Edward Durham, of London, England.

Mr. DURHAM said he desired, at the outset, to express the pleasure he felt in coming here amongst his American confrères and meeting with such a cordial reception.

The subject of intestinal obstruction was not altogether strange to him. There is no class of cases more dangerous, more serious, and which more urgently call for prompt, skillful, surgical treatment than cases of intestinal obstruction. He could most cordially agree with Dr. Senn that a surgical operation should be performed as soon as the diagnosis was made; but it was exceedingly difficult to make a diagnosis in such cases. In fact, he knew of no class of cases in which the diagnosis was more difficult, and he would venture to go a step farther, and say he had seen cases from time to time in which the surgeon was justified in operating before the diagnosis was made—in other words, to operate in order to establish an accurate diagnosis. The difficulty lies in knowing what the cause of the obstruction is, and locating its seat.

With regard to the observations made by Dr. Senn, and his suggestions as to treatment, he came to America to learn and confessed that he had learned a great deal in many places, and in many ways. Insufflation with hydrogen gas was something new to him. He would like, therefore, to ask Dr. Senn what would happen in case the patient was blown up. He supposed Dr. Senn would call it a case of "blow up" then. He could not see why hydrogen gas on account of its particular lightness should be better for insufflation than ordinary air. This he had yet to learn.

Turning to the treatment. The first thing is to determine, if possible, the seat of the obstruction. In cases of acute intestinal obstruction in which it is evident, or which we have reason to believe, that the obstruction exists in the small intestine, he believes the proper treatment to pursue, when the symptoms are severe and urgent, even though an absolute diagnosis is not made, is to open the abdomen, search for the cause, and relieve it as best we can. On the other hand, when the indications are that the obstruction is in the large rather than in the small intestine, there is not the same degree of urgency, at any rate in a very large proportion of cases, and the procedure to be adopted must depend upon the seat and cause of the obstruction. In this connection he would venture without the slightest degree of hesitation to take issue with Dr. Senn. He (Senn) stated that lumbar colotomy is an obsolete operation. Now, he would beg to say it is not obsolete; it is an operation that is performed constantly day by day in Europe. He would endeavor to do the operation in any case in which he had occasion to do so. He had dealt with cases in which he had not done the operation, and for which he had a lasting regret that he did not do so; therefore, he would unhesitatingly say that the operation of lumbar colotomy is not obsolete. Had time permitted he could quote any number of cases in which after its performance the intestinal obstruction had been relieved, the colotomy wound healed, the patient gone about and doing well.

Dr. William Miller Ord, of London, England, said he supposed he had been called upon as a physician to express the general inadequacy of that part of the profession to deal with the subject under consideration, for it usually fell to the lot of the surgeon rather than that of the physician to deal with such cases. The physician is called early in the case; and in looking back over a good number of cases of intestinal obstruction the function of the physician mainly ceased when he has come to the conclusion that the case on the one hand is what has been called by our American confrère (Senn) "dynamic" or mechanical obstruction. He thought there was a good deal of difficulty attending the diagnosis, so much so that in some instances there is absence of indication of local pain, of fever, or of anything like bloody discharge from the bowel, that one hesitates as to the decision. Perhaps the members of the Congress would think him a little wanting in self-reliance if he should adopt the views of the first speaker (Fitz), rather than those of the second (Senn) and Mr. Durham, and claim a little time. He thought physicians had seen a good many cases of intestinal obstruction in which there had been either the simple absence of the passage of the fæces, or there had been a good deal of pain and vomiting, where everything pointed strongly to intestinal obstruction, yet where no tumor nor any amount of tenderness could guide one as to the exact locality of the obstruction; that in some cases the use of opium or other narcotic, or the use of injections, had been followed by complete relief. He had been present on several occasions when a man had been examined over night with an obstruction, where the physician could find no definite sign of any local obstruction, and where he had arranged with the surgeon and his assistants to come the next morning to operate, and where either from the influence of narcotics or injections, shortly before the operation was to be performed, nature had given relief. He would agree with all the speakers that when there was satisfactory evidence that a mechanical obstruction existed, sooner or later the case must go into the hands of the surgeon, and the delay should be only a reasonably short one.

Professor ANNANDALE, of Edinburgh, Scotland, regretted to say that he had only heard part of the papers and discussion, and was therefore unable to deal with the subject in a methodical way.

In the treatment of intestinal obstruction it was very important to divide the cases into two classes, acute and chronic. With reference to acute cases, he thought it was perfectly right that everything that medicine could do should be tried, but not tried too long. As soon as it had been fairly tried, say forty-eight hours, and the symptoms are urgent, then he would say the case belongs to the surgeon, and the sooner he opens the abdomen the better.

In regard to chronic cases, he thought the surgeon might wait until the symptoms had become acute, then operate at once.

He had listened attentively to what Mr. Durham had said regarding lumbar colotomy. His experience with that operation had been such, and more particularly in cases of cancer of the rectum, that he preferred inguinal colotomy. He meant by this the performance of colotomy in the left inguinal region, because he had found it was not a more serious surgical procedure as regards risk, and was certainly more simple in a large majority of cases.

Professor FITZ had nothing new to add in closing his part of the discussion.

Professor SENN said that all would agree with him in studying English medical literature, text-books, and hospital reports, that English surgeons were exceedingly conservative. The time had long gone by when any theory or method of operation should be accepted as final, safe, and applicable, unless it had been thoroughly tried.

He could assure our distinguished guest (Mr. Durham) that hydrogen gas when injected into the intestinal canal never forms explosive compounds; that atmospheric air is never present in the intestinal canal; that the procedure has been tried more than a hundred times, and has proved thoroughly safe and infallible at the bedside. In one of the first experiments made he had to submit as (a victim) to the experiment, and would never have submitted to such a suicidal thing until its safety had been thoroughly tested on animals.

### SECOND DAY.

Professor ROSWELL PARK, of Buffalo, read a paper on "Cerebral Localization in its Surgical Relations." The essay was devoted principally to the surgical aspects of the subject, and omitted consideration of those cases in which operation is dictated by a study of the subjective rather than of the objective features.

*Cerebral Topographical Anatomy.*—The areas which most concern the surgeon are those which cluster around the fissure of Rolando. A few bony prominences deserve attention in this connection—that at the point of the nose known as the glabella; the external occipital protuberance known also as the inion; the point of the vertex half way between these two—the bregma; the external angle of the orbit, the tip of the mastoid process, and the lower border of the alveolar process of the upper jaw. The fissure of Rolando has its upper end about five centimetres back of the bregma, but does not run quite in the middle line; its lower end lies about half a centimetre behind the auriculo-bregmatic line, and a little above an imaginary line projected backward from the superciliary ridge; thus, the lower end of this fissure will be found about six centimetres above and a little behind the external auditory canal, or about an inch behind the bifurcation of the fissure of Sylvius. In regard to the convolutions, it must be stated that lesions of the dura-mater overlying motor areas are not always to be distinguished from lesions in the cortex beneath. It is enough for the surgeon that a lesion of some kind can be located with reasonable accuracy. It matters not whether this is an old, irritative lesion, an acute suppurative process between the bone and the brain, or an abscess or tumor of the brain itself. The indication for exploration is just as strong in either case.

*When and Where can one Trephine with Safety?*—The safest rule is to first apply the trephine over those areas which do not overlie large vascular channels. Afterward, the opening may be extended in any direction, and to any required extent. The greatest hesitation is with regard to opening one of the sinuses. Two dangers attend such an accident: one, fatal air embolism; the other, profuse hæmorrhage. The former danger is almost a theoretical one, and the other may be overcome by plugging the sinus or closing its wound with a fine needle and suture.

*Cerebral and Cerebellar Abscess.*—Bergmann has shown that abscess of the brain has but one result—death, and that the surgeon's knife offers the only relief. So far as we know, there is no such thing as idiopathic abscess of the brain; it is always the result of some external wound of the head, or some extension from diseased surrounding bone. The only exceptions to this statement are to be found in the case of pyæmia or tuberculous abscess. The symptoms of deep brain abscess may be divided into three groups, according to causes: 1. Those which are inseparable from indications of suppuration. Such are those disturbances which may follow any deep-seated foreign body. 2. Symptoms of increased intra-cranial pressure and of disturbed relations. 3. Special symptoms by which the locality of the disturbance may be ascertained so long as the gray matter is undestroyed; the collection of pus may assume large dimensions, and still no intense motor symptoms appear. Local elevation of temperature over the abscess is a symptom of importance when present, but its absence need not negative a diagnosis, if made on other grounds. Wernicke has stated that there is a peculiar disturbance of speech which points to lesion of the temporal region. This is the confusion of correct with incorrect words. In the general diagnosis of cerebral abscess, it is to be remembered that there usually is a latent period which may continue for an indefinite period. The stage of active symptoms is usually ushered in by more or less headache, slight rise in temperature; local or motor symptoms can only be expected when the abscess is in the motor area of the brain.

*Dangers of Operation.*—The principal immediate dangers are two—hæmorrhage and œdema. Hæmorrhage from the pia or from the brain-substance is usually readily controlled, but disastrous hæmorrhage may occur from unexpected sources. When there is bleeding a temporary tampon of iodoform gauze may be applied. The dural and skin flaps are laid over this and an absorbent dressing applied. At the end of forty-eight hours this may be removed and sutures inserted.

The second danger, that of acute brain œdema, may be brought about either by increase of intra-arterial pressure or by obstruction of the venous channels of escape. Under this accumulation the brain becomes more sodden.

The author had collected reports of sixty-three cases, which were presented in summary and in tabular form; seventeen of these terminated fatally, although only five of these deaths could properly be attributed to the operation; fifteen of the cases were abscesses, subdural or subcortical. In eleven cases the lesion was a tumor, exclusive of tubercular nodules. Of cysts, properly speaking, there were twelve. The twenty-five other cases were of a miscellaneous nature. In three cases the true character of the lesion was not revealed during the operation, and was only discovered at the autopsy. In two cases, in which no palpable or visible lesion was discovered at the time of operating, the symptoms which led to the performance of the operation were nevertheless relieved, though nothing but careful exploration was practiced.

Of the sixty-three operations, seventeen were performed by American surgeons. Those who have themselves operated more than once are, with the number of their operations: Macewen, 12; Horsley, 11; Bergmann, 4; Weir, 3; Keen, 3; and Park 3.

Professor CHARLES K. MILLS, of Philadelphia, read a paper on "Cerebral Localization in its Practical Relations." In his introductory remarks, the speaker referred to the fact that from the clinical observation of practical physicians sprang the conceptions out of which developed the science and art of cerebral localization. Allusion was made to the discoveries of Bouillaud, and of Broca, on speech localization; to the announcement by J. Hughlings Jackson, in 1864, that certain convolutions superintended the delicate movements of the hand which were under the immediate control of the mind; and to Hitzig's researches having originated from his observing certain ocular movements during galvanization of the heads of his patients. Brief reference was made to the history of American work in localization; to the investigations, in 1874, of the New York Society of Neurology and Electrology; to Putnam's discovery that irritation of the white matter beneath definite cortical centres produced movements similar to those caused by irritation of the centres themselves; to the labors of Wood and Ott on the heat centres, and to the light thrown by these investigations upon the mechanism of fever and the action of drugs upon different forms of high temperature.

Trephining for cases of insanity, particularly when guided by the rules of localization, was briefly considered. Two of the recent cases of brain operation, reported by Bennett and Gould and by Macewen, were cited as possibly opening a new field for surgical interference in insanity; the excision of cortical areas as a method of treatment when certain subjective phenomena, such as hallucinations of sight or hearing, can be given a local habitation in the brain.

The practical conclusion was that the neurologist and surgeon must depend upon motor symptoms alone in fixing the site for operation in cases where the motor lesions were definite. When positive sensory symptoms were present, they might sometimes serve to aid in locating more exactly the position for operation, but the data were not sufficient for positive reliance.

The question of morphological peculiarities of the human brain was briefly alluded to as having some practical bearing upon the subject under discussion. The position of the so-called angular gyre and aberrations in the parieto-occipital region were more particularly discussed. Even the fissure of Sylvius, the central and parieto-occipital fissures sometimes present considerable variation; but, as a rule, such aberrations were not confusing in operating on the motor region after the methods of Broca, Thane, and others.

Professor MOSES ALLEN STARR, of New

York, read a paper on "Cerebral Localization in Reference to Aphasia."

It is evident that cerebral surgery has a great future, but is dependent on neurology for its guide. The burden of responsibility for future progress rests upon physicians, for diagnosis must precede operation.

The great discoveries in cerebral localization made in the past have been reached by means of the collection and analysis of large numbers of cases of localized disease in man, rather than through physiological experiment. Future advance must be in the same line. Hence the importance, too much overlooked in this country, of recording carefully every case of cerebral disease. And to be properly recorded it must be carefully examined. This is especially necessary in cases of disturbance of speech.

The history of aphasia presents three epochs: First, that of Broca, in which the fact was established that lesions of the third frontal convolution on the left hemisphere produced aphasia. Secondly, that of Wernicke, in which a distinction between sensory and motor aphasia was drawn, and the former was shown in a few cases to be due to lesion of the first temporal convolution. Thirdly, that of Charcot, in which the four mental elements of speech were carefully separated. Charcot says, "A word is a complexus; in it we can discover, in persons of education, four distinct elements: the auditory memory-picture, by whose means we are able to grasp the sense of words heard; the visual memory-picture, which enables us to comprehend the words written or printed; and also two motor elements—the motor memory of articulation, and the motor memory of writing; the first developed by the repetition of movements of the tongue and lips necessary to pronounce a word, the second by the practice of motions of the hand and fingers necessary for writing." Each of these memories being distinct can be lost. The result is disturbance of speech whose forms vary. The loss of visual memories produces verbal amnesia and word-blindness; the loss of auditory memories causes word-deafness; the loss of motor memories of writing results in agraphia; the loss of motor memories of pronunciation produces motor aphasia. Individuals differ largely in the degree of cultivation of each of these memories, and hence suffer differently when affected by their loss; *e. g.*, the literary man presenting far more symptoms than a common laborer, when his memories of things read are lost. Another fact of importance is the independence of speech and thought. Aphasics may retain their musical faculties and may sing when they cannot talk. Thinking, though largely done by the aid of speech, is not dependent upon it. We have memory-pictures of the shape, form, or sound and odor of objects, independent of their names, and unless these are intact in the brain the perception of the object does not produce recognition of its nature or use, and does not awaken the memory of its name. The condition termed apoaxia is found, with aphasia, in some cases, but not in all.

Turning from clinical distinctions to pathology, the localization of the various memories necessary to speech was discussed. Motor aphasia is produced by lesion in Broca's centre, or in the tract from that centre to the cranial nerve-nuclei. If it is due to lesion in the latter, it is temporary and accompanied by other local symptoms. The situation of the lesion producing motor agraphia is uncertain. Word-deafness is due to lesion in the first temporal convolution, and is associated with word-blindness when the lesion extends to the supramarginal convolution and angular gyrus. All the cases of pure sensory aphasia in which the lesion was limited to these parts (forty-one in number) were collected, and a chart of the brain was shown to support the localization stated. The condition of apoaxia was shown to accompany lesions situated in or beneath the angular gyrus.

Dr. David Ferrier, of London, England, in opening the discussion on the above papers, said he took special pride and satisfaction in the fact that this subject had been assigned such an important place in this great gathering of the profession in this country.

He had long cherished the idea that the determination of the function of the brain would in time lead to successful treatment by surgery of some of the most distressing ailments of our fellow creatures. There is a great future for cerebral surgery. While there have been many successes, yet there have been some failures. He alluded more particularly to cases of Jacksonian epilepsy. The discharging lesion had been removed in many of these cases without permanent cure. There is yet much to be learned in regard to the functions of the brain and in regard to the diagnosis of cerebral disease.

Mr. VICTOR HORSLEY, of London, England, described briefly the results of his experiments upon the motor region. He believed that here three functions were clearly represented: First, the representation of the so-called tactile sense; second, representation of the so-called motor sense; third, the great representation of movement. It is found that, morphologically, the large cells in the fourth layer are concerned in the representation of movement, and he could not understand why we should not allot to the small cells in the upper layer the representation of sensation. He had divided the motor region into minute areas, and studied the effects of irritation of these separate areas. He had found that the representation for any part was not limited to one minute portion of the brain, but that there was a focal point where it was strongest, and then it graduated as we passed outward. In his different experiments he had met with certain points of difference. These were attributable to the employment of different species of monkeys. He now uses only the bonnet monkey, and for all practical surgical purposes, the results are applicable to man.

Experiments performed during the past summer had enabled him to prove that the convulsions of so-called Jacksonian epilepsy were solely due to the cortex, and not at all dependent upon the spinal cord, or upon the bulbar spinal system.

Professor ROBERT F. WEIR, of New York, had, since 1883, operated in ten cases of brain surgery—three times for tumor, three for abscess, twice for hæmorrhage into the cerebrum where there was no external injury to indicate its locality, once for epilepsy, and once for cerebral pain.

Professor KEEN has removed a tumor weighing over four ounces, with recovery, and Mr. HORSLEY has removed with success a tumor weighing four and a half ounces from a patient in a state of coma.

### THIRD DAY.

The closing session of the Congress was held in the Army Medical Museum.

Dr. BILLINGS, in delivering the President's address, said the prominent characteristics of the great majority of the societies composing the Congress is that their members have, as a rule, been chosen because they have either made some valuable contribution to medical literature, or have in some way rendered aid to the profession; in other words, they are supposed to be men whose labor and thought have not been confined to their own interests, or to those of their own patients. It may, therefore, be assumed that physicians are all interested in medical science, not merely as a means of giving new modes of diagnosis, or of treatment, but also for its own sake, for the sake of knowing and for the pleasure of investigation.

He was here as the representative of the medical department of the General Government, which has need of the best knowledge of all the specialties, and is beginning, in its turn, to do something for each.

Within the last twenty-five years the General Government has, in its turn, done something for medicine and for physicians by founding and maintaining a medical library and museum in Washington, under the direction of the Medical Department of the Army.

The necessities of modern progress in anatomy, physiology, and pathology, have led to the creation of medical museums in all parts of the civilized world. In most of the continental capitals these are connected with universities supported by the state. The medical museum should possess a series of specimens showing the normal anatomy of the domestic animals, or of animals used in experimental pathology, pharmacology, or physiology, as a basis for comparison with abnormal or pathological specimens derived from the same animals.

Elaborate dissections under alcohol, mounted in opaque dishes with flat glass covers, and sections of frozen bodies similarly mounted, are what the student and practitioner most desire to see. In our museum there are some excellent specimens of this kind prepared under the direction of Professor His of Leipzig, of Professor Cunningham of Dublin, and by our own anatomist, Dr. Wortman. The annual appropriation for the museum at present is $5,000. This is sufficient, except that the printing of the catalogue must be an extra charge; but the medical profession should see to it that the amount is not reduced in the rhythmic spasms of partial economy with which some of our statesmen are afflicted.

Speaking in behalf of the Army Medical Department, and for the dead as well as for the living, who have been charged with this work, he could truly say that they had been very proud of their charge, and that they had done their best to make the museum and library such as a great profession and a great nation have a right to demand.

At the conclusion of the address a reception was given to the members and guests of the Congress and their ladies.

